# Cycle threshold of SARS-CoV-2 RT-PCR as a driver of retesting

**DOI:** 10.1038/s41598-024-52984-7

**Published:** 2024-01-29

**Authors:** Robert Markewitz, Justina Dargvainiene, Ralf Junker, Klaus-Peter Wandinger

**Affiliations:** Institute of Clinical Chemistry, University Hospital of Schleswig-Holstein, Arnold-Heller-Straße 3, 24105 Kiel, Germany

**Keywords:** Infectious-disease diagnostics, Microbiology, SARS-CoV-2

## Abstract

SARS-CoV-2 RT-PCR is a critical and, at times, limited resource. Frequent Retesting of patients may strain testing infrastructure unduly. Recommendations that include cycle threshold (Ct) cutoffs may incentivize early retesting when the Ct value is reported. We aimed to investigate patterns of retesting in association with initial Ct-values. We performed a retrospective analysis of RT-PCR results (including Ct-values) for patients from whom ≥ 2 samples were collected within 14 days, the first of which had to be positive. We calculated absolute and baseline-corrected kinetics of Ct-values over time, as well as the median initial Ct-values in dependence of the timing of the first retesting and the time until RT-PCR negativity for SARS-CoV-2. Retesting after an initial positive SARS-CoV-2 RT-PCR was most commonly performed on day 7, with patients being retested as early as day 1. The majority of patients retested within 14 days remained SARS-CoV-2 positive in the RT-PCR. Baseline-corrected Ct-values showed a quasi-linear increase over 14 days since the initial positive result. Both the timing until the first retesting and until RT-PCR negativity were inversely correlated with the initial Ct-value. The timing of retesting after a positive SARS-CoV-2 RT-PCR appears to be significantly influenced by the initial Ct-value. Although it can be assumed that Ct-values will increase steadily over time, strategies that rely on rigid Ct-cutoffs should be discussed critically, not only because of methodological caveats but also because of the strain on testing infrastructure caused by the incentive for early retesting that Ct-values apparently represent.

## Introduction

In the course of the COVID-19 pandemic, the estimation of the viral load of SARS-CoV-2 using the cycle threshold (Ct) of the RT-PCR has become a common surrogate marker of infectiousness. Despite important caveats concerning the uncritical equation of Ct-value and viral load^1,2^, and even though it is not recommended in any guideline by leading organizations such as the CDC, the ECDC or the WHO, it is our personal experience that many clinicians still employ rigid thresholds, most commonly Ct > 30–34^3–5^, for clinical decision-making concerning discharge or isolation of patients. While methodological concerns regarding this practice have been voiced abundantly^1,2^, the underlying assumption is that Ct-values increase linearly as the viral load is steadily diminished by the immune system’s response during the individual course of COVID-19. In our experience, this assumption, in front of Ct-based rules concerning quarantine or discharge of patients, leads to frequent retesting in some cases, with the goal of obtaining a result that meets the required Ct-criteria (or a negative result), especially in patients with a relatively high initial Ct-value. Retesting that is motivated mainly by Ct value requirements may place an undue burden on testing infrastructure, especially in times of a global pandemic such as the world has just witnessed. Therefore, we aimed to examine, from our own data, patterns of retesting of COVID-19 patients, especially with regards to the Ct values obtained.

To this end, we retrospectively analyzed RT-PCR results and associated Ct-values from the daily routine of a high-throughput clinical laboratory. Included in this analysis were all results from individuals of whom at least two SARS-CoV-2 RT-PCR tests (nasopharyngeal swabs) within 14 days were performed (at least the first of which had to be positive).

## Methods

### Study cohort and sample characteristics

Samples to be included in the subsequent analyses were selected following this procedure: For each individual included in our data base who had been tested positive for SARS-CoV-2 via RT-PCR at least once, the first (i.e. in temporal order) positive sample was identified. All subsequent samples, be they positive or negative, that were collected within 14 days after this initial positive sample of each individual were included in the analysis.

As a retrospective evaluation of anonymized data, the study was ruled exempt from institutional board review by the ethics committee of the University of Lübeck (AZ: 2023-482) and informed consent was not required. All research was performed in accordance with relevant guidelines and regulations, including the declaration of Helsinki.

### RT-PCR testing

All Ct-values are derived from the amplification of the E-gene of SARS-CoV-2 using the Cobas 6800 System (Roche, Switzerland) and were obtained in the same laboratory (central core laboratory, University Hospital Schleswig–Holstein, Kiel, Germany).

### Statistical analysis

Correlations between two continuous variables (such as Ct-values and days since initial positive result) were examined calculating Spearman’s rho. Baseline correction for Ct-values was achieved by calculating a ratio dividing each Ct-value of a positive follow-up RT-PCR by the initial Ct-value of this individual patient. Statistical significance was assumed for *p* < 0.05. All statistical analyses were performed using the open-source software for statistical computing and graphics R (version 4.1.0) with the integrated development environment RStudio (Version 1.4.1717)^6^.

## Results

**Figure 1:**
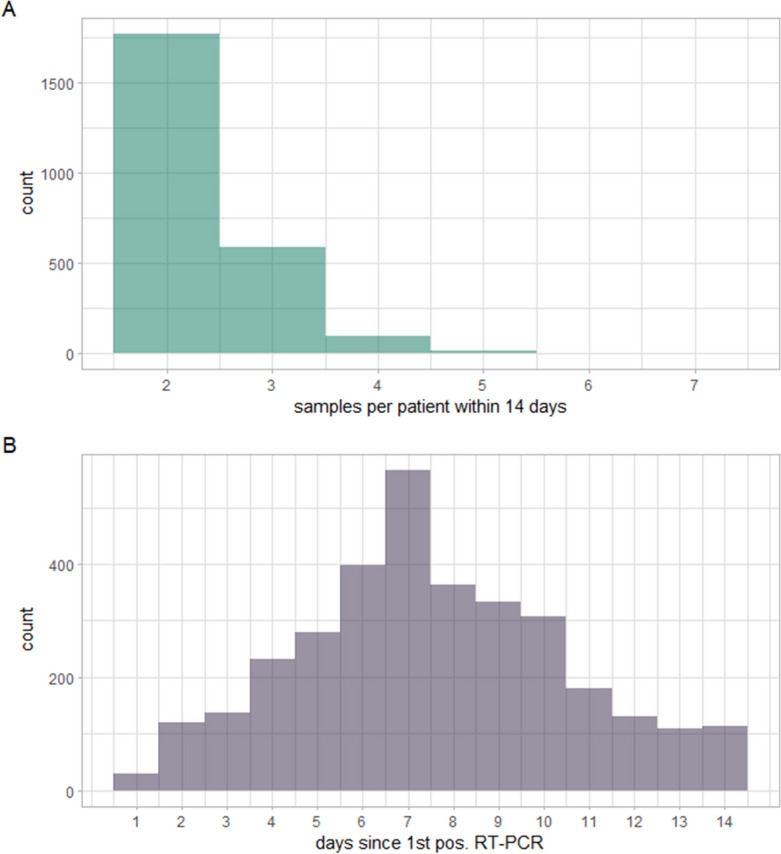
Histograms of the frequency of both samples per patient within 14 days since the first positive SARS-CoV-2 RT-PCR (**A**) and of the day of retesting since the first positive SARS-CoV-2 RT-PCR (**B**).

In all, 5754 RT-PCR samples from 2484 individual patients, collected between November 7th, 2020 and May 22nd, 2023. Of all RT-PCR samples, Ct-values were available for 5483 samples from 2483 individual patients. The median number of RT-PCR samples per patient was 2 (range: 2–7), the most frequent day of retesting since the initial positive result was day 7. The frequency of retesting and the distribution of the time of retesting for both RT-PCR is visualized in Fig. 1 (panels A and B, respectively). The RT-PCR samples were collected in four different testing facilities for healthcare professionals (HCP) as well as one emergency room and one anesthesiologic outpatient clinic.

**Figure 2:**
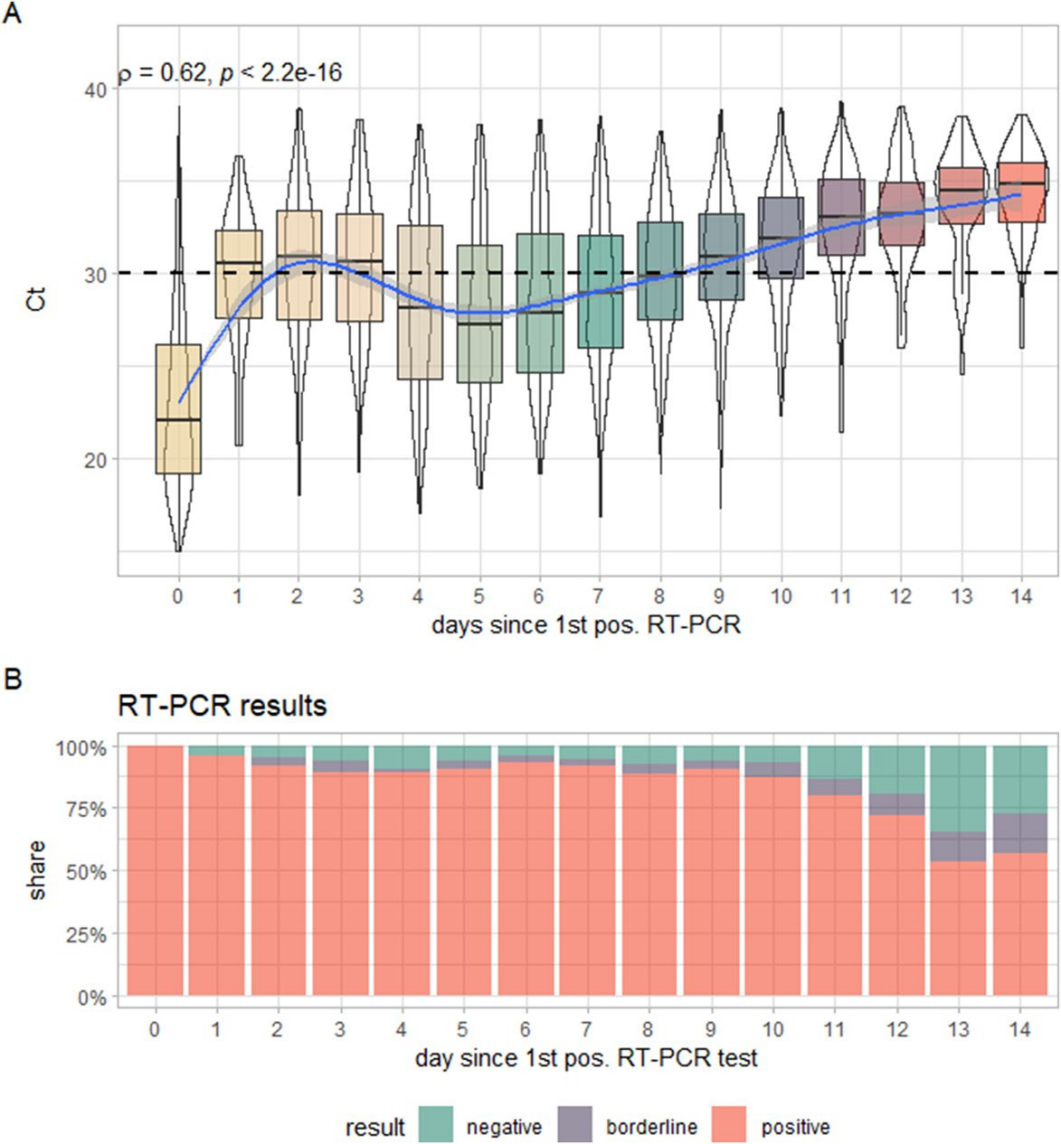
(**A**) Average Ct-values per day since first positive SARS-CoV-2 RT-PCR for all samples. Spearmans’s rho including p-value is indicated in the upper left-hand corners; blue lines represent smoothed means with 95% confidence band indicated in grey. The dotted horizontal line represents an exemplary cut-off of Ct > 30. (**B**) Shares of positive, borderline and negative SARS-CoV-2 RT-PCR tests per days since the first positive test.

The cumulative analysis of these samples shows a statistically highly significant correlation of medium effect size (ρ = 0.62; *p* < 0.0001, Fig. 2A) between Ct-values and days since the first positive SARS-CoV-2 RT-PCR. However, this correlation is non-linear, with Ct-values quickly increasing above 30 until an intermediary peak at days 1–3 before decreasing again until an intermediary nadir at day 5 only to cross the line of Ct > 30 again at day 8. Positivity rates of SARS-CoV-2 RT-PCR remain high almost throughout the first 14 days after the first positive result and only drop noticeably after day 10 (with a relatively high share of borderline results at days 10–14) and reach a positivity rate of 54.4% at day 14. At day 7, the day of the most frequent retesting, we found a positivity rate of 91.7% in our cohort (Fig. 2B).

**Figure 3:**
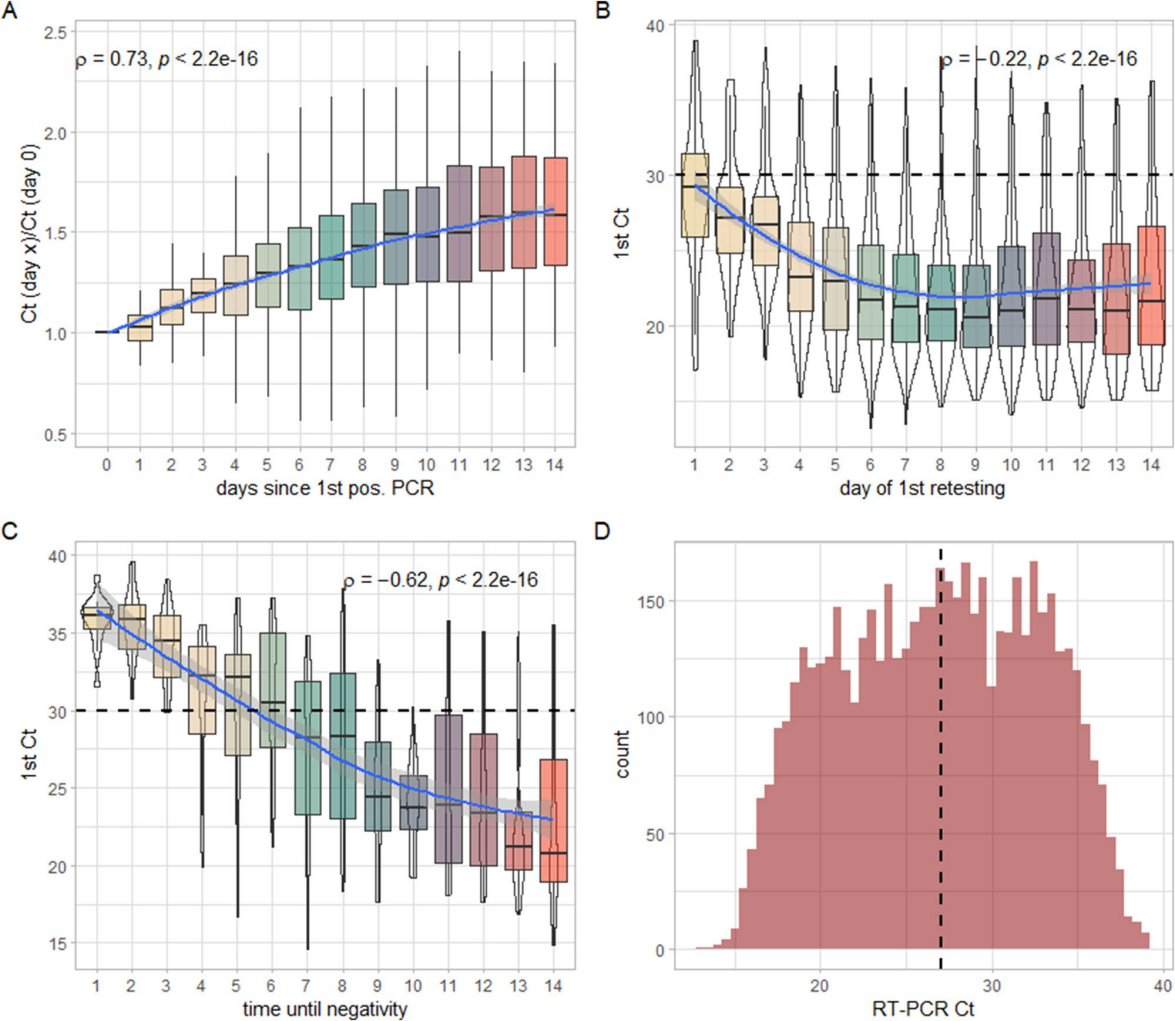
(**A**) Baseline-corrected average Ct-values per day since first positive SARS-CoV-2 RT-PCR for all samples. Spearmans’s rho including *p*-value is indicated in the upper left-hand corners. (**B**) Average initial Ct-values as a function of the day of the first retesting for SARS-CoV-2 via RT-PCR. Spearmans’s rho including *p*-value is indicated in the upper right-hand corners. (**C**) Average initial Ct-values as a function of the individual latency between the first positive and the first negative RT-PCR result for SARS-CoV-2. Spearmans’s rho including *p*-value is indicated in the upper right-hand corners. (**D**) Histogram showing the distribution of Ct-values in all examined samples. The dashed vertical line represents the median Ct-value. Blue lines in (**A**–**C**) represent respective smoothed means with 95% confidence band indicated in grey. The dashed horizontal lines in (**B**) and (**C**) represent an exemplary cut-off of Ct > 30.

While the association between absolute Ct-values and days since the first positive result is, as mentioned, non-linear, the picture is completely different if a baseline correction is performed by calculating Ct ratios by dividing the Ct-value of each follow-up RT-PCR by the Ct-value of the first positive sample. Here we see an almost completely linear increase of the Ct-values relative to the baseline value over time with a highly significant correlation of strong effect size (ρ = 0.73; *p* < 0.0001, Fig. 3A).

To account for this apparent discrepancy between the kinetics of absolute and relative Ct-values over time, we compared the initial Ct-values by the amount of days elapsed between the first positive result and the first retesting via RT-PCR. We found that those who returned in the very early days after their first positive result for retesting had significantly higher initial Ct-values than those who returned for retesting at a later date. The negative correlation between the initial Ct-values and the day of retesting was statistically highly significant, albeit with a small effect size (ρ =  − 0.22; *p* < 0.0001, Fig. 3B).

We also compared the initial Ct-values in dependence of the amount of days elapsed between the first positive and the first negative RT-PCR result. Again, we found a highly significant negative correlation, of medium effect size (ρ =  − 0.62; *p* < 0.0001, Fig. 3C), indicating that lower initial Ct-values lead to a significantly longer time until the patient is tested negative, and vice versa. It must be noted, however, that, as the positivity rates show, a large proportion of our cohort tested positive throughout the first 14 days after the initial positive result.

Overall, the distribution of Ct-values of all positive samples approaches a normal distribution with a median of 27 ± 7.06 (Fig. 3D).

Analyzing the number of samples tested per patient within 14 days after the initial positive result, we found no correlation of meaningful effect size between number of samples and the initial Ct-value (ρ =  − 0.062, *p* = 0.0015).

## Discussion

Our results show that retesting for SARS-CoV-2 within the first 14 days after an initial positive result was a frequent occurrence in our laboratory. There appears to have been two putative main causes for retesting: Obtaining a negative result one week after the initial positive result (as witnessed by the fact that day seven was the most common day for retesting), which was a common recommendation of hygiene experts in German hospitals, and obtaining a Ct-value that meets certain criteria (e.g. > 30), as witnessed by the fact that a relevant number of individuals returned as early as one day after the initial positive result. The assumption that early retesting was at least to some degree motivated by Ct-values is corroborated by our finding that initial Ct-values are inversely correlated with the amount of days elapsed at the first retesting. Frequent retesting in short intervals might, of course, be used to identify conversion to RT-PCR negativity as early as possible (especially if it is guided by the initial Ct-value) in order to stop isolation or quarantine for the individual patient. But, as our further results show, conversion to RT-PCR negativity within a few days (and even within the first 14 days) of the initial positive result is a rarity and happens mostly if the patient had an initial Ct-value > 35, which is considerably higher than the median initial Ct-value of patients who returned after just one day in our data.

We also found that baseline-corrected Ct-values increase in a quasi-linear fashion and that a shorter amount of time from the initial positive test until the first negative test is associated with higher initial Ct-values. Both of these findings could be used to argue in favor of Ct-guided RT-PCR retesting. However, the positivity rates of the RT-PCR over time since the initial positive result also show that the overwhelming majority of SARS-CoV-2 RT-PCR tests performed in our cohort within the first 14 days after the initial positive test still yielded a positive result. On the most frequent day for retesting (day 7), this rate was at a staggering 90.5%. Therefore, while a higher initial Ct-value is apparently associated with a shorter time until SARS-CoV-2 RT-PCR negativity, this is relevant only for a minority of cases within the first 14 days after the initial positive result.

All in all, it is reasonable to conclude that the patterns of retesting for SARS-CoV-2 in our cohort were significantly shaped by the knowledge of the Ct-value of previous SARS-CoV-2 RT-PCR tests. In our view, this should at least receive scientific scrutiny, which was the aim of this study. The fact remains that nasopharyngeal swabs are, essentially, no quantifiable samples in the traditional sense and, furthermore, Ct-values are no standardized, quantitative values. Specifically, Ct-values may vary significantly between different genes examined^7,8^, and, within the same gene, between different strains of the same pathogen^9,10^ as well as between assays from different manufacturers^7^, and, within an assay from a single manufacturer, between different runs and different instruments^1^. Most importantly, however, Ct-values are also dependent on the quality of the collection of the swab^11^. Attempts at absolute quantification of SARS-CoV-2 RNA copies from have been undertaken^12,13^, but have not gained widespread use. While evidence has been gathered that infectivity of SARS-CoV-2 is inversely correlated with Ct-values^14^, it is therefore evident, that a definitive, single Ct cutoff for infectivity cannot be defined. As a consequence, there is somewhat of a controversy whether Ct-values should even be reported, with the general consensus, especially among clinicians being that they should, even given all abovementioned caveats^15^.

Our data, however, add a new dimension to this debate: Reporting Ct-values did apparently significantly influence the timing of retesting. In our view, it is fair to question how sensible it is to test the same individual multiple times within a few days, in order to obtain a desired result, the probability of which is estimated by an initial Ct-value. Not only does this strategy add to the significant strain that SARS-CoV-2 has put not only on the testing infrastructure (laboratories and swab collection sites) and the economy (RT-PCR tests being costly and reagent having been in short supply, especially at the beginning of the pandemic), but it also motivates patients to leave quarantine at an early point (while possibly still infectious) to get retested, despite the fact that an initial high Ct-value might also be the result of poor sample collection. On the other hand, frequent and early retesting might allow to end isolation or quarantine for individual patients, which is an important goal to achieve, considering the adverse effects that isolation has on hospitalized patients^16^ as well as the cost and heightened workload it causes for healthcare facilities. However, this goal cannot be reasonably achieved, in our view, by the reporting of “raw” Ct-values, due to the abovementioned caveats. A valid and evidence-based solution is the semi-quantitative reporting of an approximate viral load as being either above or below the threshold of 10^6^ copies per milliliter^17,18^. If this strategy is chosen, it should be emphasized, however, that for its implementation, a reference sample with a known viral load of exactly 10^6^ copies per milliliter needs to be included in every PCR run. It should furthermore be added that even below this threshold, shedding of culturable virus particles and therefore infectiousness may still be possible (if less likely than above this threshold)^17,18^, which is why it should always be interpreted in synopsis with the temporal course of the individual case of COVID-19. Infectivity of an individual patient may never be completely excluded via RT-PCR of nasopharyngeal swabs, which is why the implementation of every strategy of deisolation based on it will always include a tradeoff between the benefits and risks of deisolation, as well as it should include, as we have tried to show, a focus on the reasonable use of laboratory resources.

It is true, of course, that the patterns of retesting described above are not, primarily, the consequence of reporting Ct-values, but rather the consequence of guidelines and directives that have relied on Ct-values for recommendations on quarantine and discharge of patients. In the course of the pandemic, regulatory bodies have mostly reversed this course and recommended quarantine for a variable amount of days after a positive result^19–21^, but the possibility to end quarantine on the basis of a negative RT-PCR result, that is still contained in some guidelines^20,21^, will likely continue to incentivize early retesting, depending on the initial Ct-value.

As a limitation, we have had no information on the patients’ symptoms at the time points of the individual sample collection. As a consequence, neither did we have information on the interval between onset of symptoms and sample collection. As witnessed by the wide range of initial Ct-values, our cohort was likely very heterogenous, with initial sample collection in all stages of infection with SARS-CoV-2.

In conclusion, while our data do show that Ct-values rise linearly over time and that higher initial Ct-values are associated with a shorter time until RT-PCR negativity, we could also detect that reporting of Ct-values leads to specific patterns of RT-PCR retesting, the meaningfulness of which should at least be discussed critically. If nothing else, it should serve to inform us for possible future pandemics, at the beginning of which testing capacities may again be limited and testing strategies should focus on a sensible use of the available resources.

## Data Availability

The datasets analyzed during the current study are available from the corresponding author upon reasonable request.
